# A generalizable and interpretable model for mortality risk stratification of sepsis patients in intensive care unit

**DOI:** 10.1186/s12911-023-02279-0

**Published:** 2023-09-15

**Authors:** Jinhu Zhuang, Haofan Huang, Song Jiang, Jianwen Liang, Yong Liu, Xiaxia Yu

**Affiliations:** 1 School of Biomedical Engineering, Shenzhen University Medical School, Shenzhen University, Shenzhen, Guangdong 518055 China; 2 Department of Biomedical Engineering, Hong Kong Polytechnic University, Hong Kong SAR, China; 3 Department of Intensive Care Unit, Shenzhen Hospital, Southern Medical University, Shenzhen, China

**Keywords:** Sepsis, In-ICU mortality, Risk stratification, Multi-source data, XGBoost, SHAP

## Abstract

**Purpose:**

This study aimed to construct a mortality model for the risk stratification of intensive care unit (ICU) patients with sepsis by applying a machine learning algorithm.

**Methods:**

Adult patients who were diagnosed with sepsis during admission to ICU were extracted from MIMIC-III, MIMIC-IV, eICU, and Zigong databases. MIMIC-III was used for model development and internal validation. The other three databases were used for external validation. Our proposed model was developed based on the Extreme Gradient Boosting (XGBoost) algorithm. The generalizability, discrimination, and validation of our model were evaluated. The Shapley Additive Explanation values were used to interpret our model and analyze the contribution of individual features.

**Results:**

A total of 16,741, 15,532, 22,617, and 1,198 sepsis patients were extracted from the MIMIC-III, MIMIC-IV, eICU, and Zigong databases, respectively. The proposed model had an area under the receiver operating characteristic curve (AUROC) of 0.84 in the internal validation, which outperformed all the traditional scoring systems. In the external validations, the AUROC was 0.87 in the MIMIC-IV database, better than all the traditional scoring systems; the AUROC was 0.83 in the eICU database, higher than the Simplified Acute Physiology Score III and Sequential Organ Failure Assessment (SOFA),equal to 0.83 of the Acute Physiology and Chronic Health Evaluation IV (APACHE-IV), and the AUROC was 0.68 in the Zigong database, higher than those from the systemic inflammatory response syndrome and SOFA. Furthermore, the proposed model showed the best discriminatory and calibrated capabilities and had the best net benefit in each validation.

**Conclusions:**

The proposed algorithm based on XGBoost and SHAP-value feature selection had high performance in predicting the mortality of sepsis patients within 24 h of ICU admission.

## Introduction

Sepsis is a heterogeneous life-threatening syndrome that affected approximately 50 million patients globally and resulted in 11 million deaths worldwide in 2017 [1]. It poses a significant burden in the intensive care unit (ICU) with high in-ICU mortality, ranging from 22 to 42% [2, 3]. Patients survived from sepsis could still suffer from long-term health consequences [2]. Timely and appropriate interventions are crucial to save their lives [4]. It is essential to identify those patients who are at a high risk of deterioration [5]. A risk stratification model could facilitate the identification of patients with different mortality risks. Those patients with a high risk of death require aggressive interventions, whereas those patients with a relatively favorable prognosis could receive more conservative management [5, 6]. In the ICU, developing such sepsis risk prediction models could better optimize the medical resources to give appropriate individualized treatments and improve patient outcomes [7].

Currently, a variety of clinical risk scoring systems are used routinely in the ICU settings to stratify patients with sepsis, such as sequential organ failure assessment (SOFA) score [8], systemic inflammatory response syndrome (SIRS) [9], and the simplified acute physiology score (SAPS) [10]. These traditional scoring systems were created mainly based on physicians’ knowledge and experience [11], and target the general patient population in the ICU, not the sepsis population specifically. Some studies have reported that these scores lacked sensitivity and specificity and performed poorly to predict morbidity and mortality in early stage of sepsis [12, 13]. The prognostic significance of quick SOFA (qSOFA) score is not specific to infection [14], and the SOFA score aims to assess the severity of organ failure rather than make predictions [15]. Up to 90% of ICU patients could meet the SIRS criteria during their stay [16]. In addition, most of these scores are calculated based on the laboratory test results that take time to obtain [7, 17]. The accuracy of the scores was attributed to the numerous variables, which also led to their complexity in the calculation [17].

Recently, machine learning (ML) techniques have become increasingly popular in the medical field due to the growing amount of electronic health data and advancements in the computer technology [18, 19]. ML can process high-complexity clinical information and use the acquired knowledge to diagnose, manage, and predict disease outcomes [19]. Patient risk stratification is one of the most wide potential applications of ML techniques [20]. Several studies have already developed ML-based risk assessment models for patients with sepsis in the ICU settings [21–23], such as Extreme Gradient Boosting (XGBoost), stepwise logistic regression, stochastic gradient boosting, and random forest (RF). These ML models were reported to have satisfactory sensitivity and specificity on the training data. However, these models are limited by their small sample sizes, few predictor variables, or old sepsis definitions [22]. In addition, the lack of external validation, poor interpretability, and non-transparency from black box issues have limited the clinical applications of these models [21, 24]. Lundberg et al. [25] proposed a Shapley Additive Explanation (SHAP) technique to overcome the black box issue during the ML model creation. Explainable ML has been successfully applied to sepsis mortality prediction [21, 23].

Therefore, in the present study, we developed an interpretable risk stratification model for the sepsis population in ICU settings based on the SHAP technique [25] and XGBoost algorithm [26] by using multi-source data. XGBoost can handle sparse and missing data [27], such as EHR data. In order to evaluate the generalizability of the proposed model and because model validation is necessary for digital health product development, the proposed model was validated in different databases and compared with the existing clinical scoring systems available in the databases (such as SOFA, SAPS, and APACHE-IV scores).

## Methods

### Databases

Data used in this study were extracted from four publicly available databases. The Medical Information Mart for Intensive Care (MIMIC-III v1.4, ICU admissions at the Beth Israel Deaconess Medical Center between 2001 and 2012, USA) [28] database was used for the model development and internal validation. The other three databases were employed for the external validation, the MIMIC-IV v1.0 (ICU admissions at the Beth Israel Deaconess Medical Center between 2008 and 2019, USA) [29], the eICU Collaborative Research Database (ICU admissions across USA between 2014 and 2015) [30], and the critical care database encompassing patients with infection at Zigong Fourth People’s Hospital (Zigong, ICU admissions between 2019 and 2020, China) [31]. Since the MIMIC-IV and MIMIC-III have overlapping timelines, duplicate patients in the MIMIC-IV were removed based on timing information. Data were stored in PostgreSQL (v13.1). The SQL scripts were developed and queried via Navicat Premium (v16.0.10).

### Study population

**Fig. 1:**
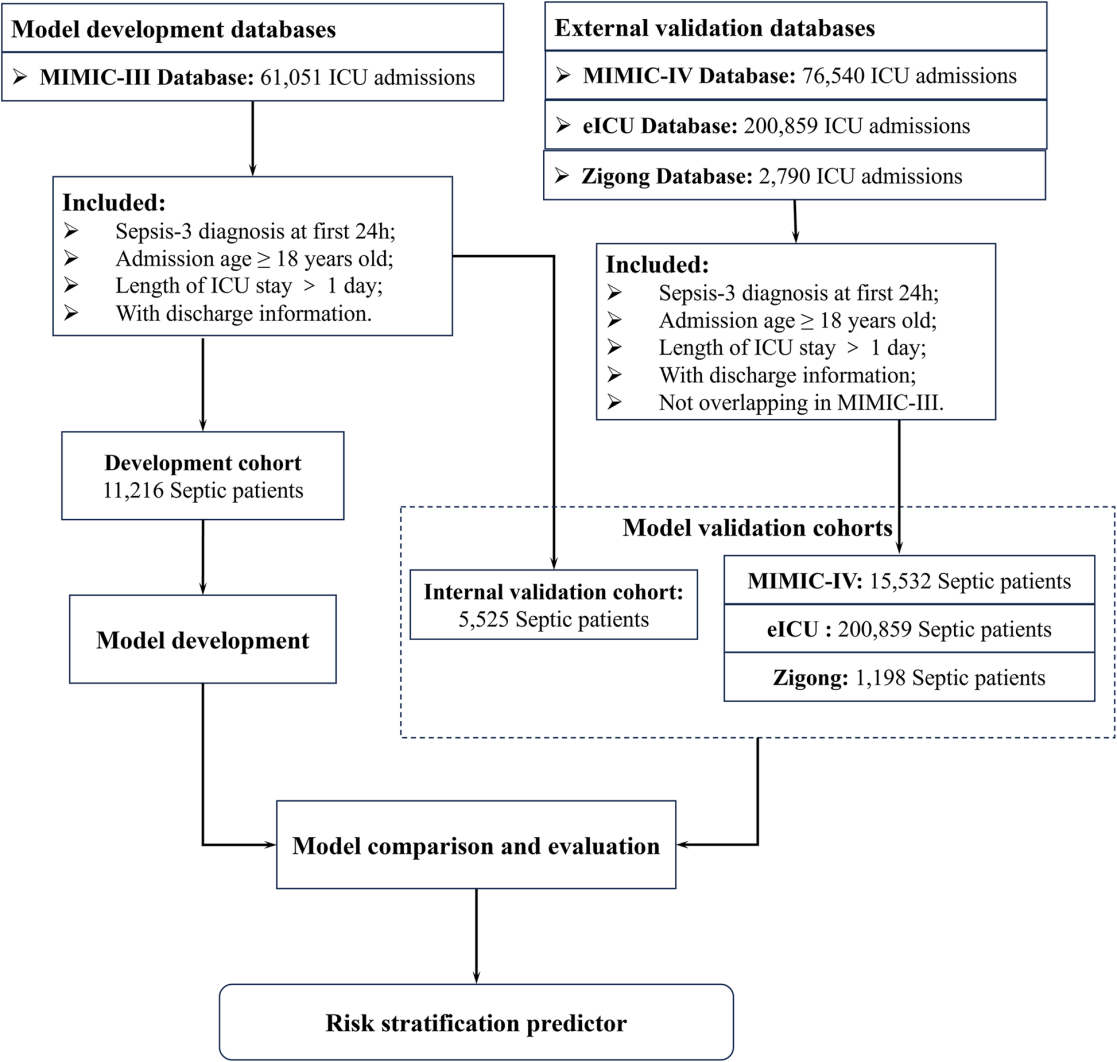
Flowchart of data processing and model development

Adult patients (ages 18 and over) were included in this study. Adult patients with sepsis at the time of ICU admission were diagnosed based on Sepsis-3 [4, 32]. The inclusion criteria in this retrospective analysis were as follows: 1) Having infection-related diagnostic records or microbiological examination and antibiotic treatment within 24 h of ICU admission; 2) the total SOFA score increased by 2 points or more. Baseline SOFA score was assumed to be 0 [4, 33]. Exclusive criteria included: 1) Missing discharge information; 2) Length of ICU stay ≤ 1 day; 3) A missing rate $\ge$ 70% in collected variables. The same inclusion and exclusion criteria were applied for all datasets. The flowchart of study design is shown in Fig. 1.

### Data pre-processing

A total of 125 variables were collected and extracted from MIMIC-III, MIMIC-IV, eICU, and Zigong databases for patients who were admitted to ICU within 24 h. These variables included demographics, vital signs, laboratory results, and SOFA score. The existing clinical score systems in the databases were also obtained, including SOFA, APACHE-IV, and SAPS scores.

For each database, all continuous variables with multiple measurements within 24 h of ICU admission were aggregated into the average value for final analysis. For numeric variables, the missing values were filled in using the median; for discrete variables, “null” was used as the default value. The clinicians checked and converted the terms and units of measurement for the features in the different databases.

### Model development and evaluation

This study applied an ML technique to create a model that could stratify the risk of in-ICU mortality in adult patients with sepsis. Model development and evaluation consisted of the following four steps.

#### Feature selection and model interpretation

Feature selection was used to eliminate irrelevant or redundant features. SHAP, as a unified framework for interpreting predictions and feature selection from tree ensemble methods [25], was applied to rank the contributions of each feature and interpret the model. In addition, the predictor selection was also referred to the opinions of four physicians and the existing clinical scoring systems. More specifically, the SHAP method was initially utilized to assess the importance of each feature in predicting events. Physicians then examined the top-ranked features based on importance scores and removed confounding factors that were not consistent with clinical practice to obtain candidate features. By repeating this feature selection process, the top 15 features selected multiple times for the pending list were used as the final modeling variables (the lower the importance, the less stable the ranking of the features).

#### Model development

For the traditional scoring system, if the corresponding standard did not propose a calculation method for the probability of in-ICU mortality, a logistic regression (LR) model of the score was established. Therefore, the LR models of the SOFA, SIRS, and Logistic Organ Dysfunction System scores (LODS) were established. Grid search and eight-fold cross-validation (CV) were used for the fine-tuning step. The best combination of hyperparameters was selected based on the nested and non-nested CV scores. These models were trained and tested using the Scikit-learn toolkit (v1.0.2) in the Python scripting language (v3.8.5).

#### Internal and external validation

The proposed model was compared with clinical score systems in four validation cohorts. The MIMIC-III population was split into training and internal validation sets (ratio 2:1) by stratified random sampling according to in-ICU mortality. In addition, three external validations were performed to assess whether the model predictions would hold true in different settings. The MIMIC-IV population was used for temporal validation, eICU for multi-center validation, and Zigong for validation in Chinese population. All numeric features were processed by Z-score normalization to reduce the impact of heterogeneity among databases.

#### Model comparison

Different ML methods were used to establish the model. A “dummy” classifier, following a simple stratified strategy [34], was used as a baseline model for comparison. Accuracy, Matthews correlation coefficient (MCC), geometric mean (G-mean), F1-score, and area under the curve (AUC) were used as the performance metrics to compare the algorithms.

#### Model evaluation

The predictive efficiencies of the model and traditional clinical scores were compared by various metrics. The area under the receiver operating characteristic curve (AUROC) and decision curve analysis (DCA) were assessed to evaluate the performance of the models. The Delong test was used to perform pairwise comparisons of the AUROC of the models. In addition, discrimination and calibration are two important aspects of model performance. The discrimination of this study was evaluated using the ROC curve, the box plots of predicted mortality, and the corresponding discrimination slopes, defined as the difference between the mean predicted mortality in survivors and non-survivors [35]. Considering that it was difficult to determine whether individual risk estimates differed between the new model and other models [36, 37], continuous net reclassification index (cNRI) and integrated discrimination improvement (IDI) were used as reclassification metrics to quantify the improvements provided by the proposed model to the traditional scoring system.

### Statistical analysis

The statistical analysis was focused on comparing the heterogeneity of different populations. Continuous variables were reported as median and interquartile range (IQR) under a normal distribution and analyzed by Kruskal Wallis rank sum test, whereas categorical variables were reported as percentages and analyzed by Chi-square test. Statistical significance was set at a *p* < 0.05. Python scripting language (v3.8.5) and R (v4.1.1) were employed for the analysis.

## Results

### Patient characteristics

A total of 16,741 patients with sepsis were extracted from the MIMIC-III population to serve as the development set. Additionally, 15,532, 22,617, and 1,198 patients with sepsis from the MIMIC-IV, eICU, and Zigong, respectively, served as the external validation sets. The characteristics of these patient cohorts are shown in Tables 1 and 2.

**Table 1:** Characteristics of included patients

Characteristics	MIMIC-III	MIMIC-IV	eICU	Zigong	*P* value
Number of patients	16,741	15,532	22,617	1,198	-
Age, years	67.00 [55.00, 79.00]	68.00 [58.00, 78.00]	67.00 [56.00, 78.00]	68.00 [55.00, 78.00]	0.080
Gender					
Male	9,352 (55.9)	9,393 (60.5)	12,153 (53.7)	737 (61.5)	< 0.001
Female	7,389 (44.1)	6,139 (39.5)	10,464 (46.3)	461 (38.5)	
Respiratory rate, breaths/min	19.00 [17.00, 22.00]	19.00 [17.00, 22.00]	20.00 [17.00, 23.00]	17.77 [15.61, 19.65]	< 0.001
Spo2, %	98.00 [96.00, 99.00]	97.00 [96.00, 99.00]	97.00 [96.00, 99.00]	98.85 [98.14, 99.38]	< 0.001
Temperature, °C	36.85 [36.41, 37.35]	36.83 [36.62, 37.12]	36.86 [36.58, 37.21]	36.88 [36.63, 37.30]	< 0.001
Heart rate, beats/min	87.00 [76.00, 100.00]	84.00 [76.00, 96.00]	88.00 [77.00, 100.00]	88.08 [74.87, 101.74]	< 0.001
SysBP, mmHg	113.00 [104.00, 126.00]	112.00 [104.00, 121.00]	112.00 [103.00, 124.00]	123.79 [113.27, 131.81]	< 0.001
Creatinine, mg/dL	1.13 [0.80, 1.93]	1.00 [0.75, 1.50]	1.00 [1.00, 2.00]	0.81 [0.61, 1.19]	< 0.001
Urine output, ml	1455.00 [827.00, 2330.50]	1490.00 [905.00, 2265.00]	1162.00 [555.0, 2020.00]	2330.00 [1075.00, 3645.00]	< 0.001
Lactate, mmol/L	1.80 [1.25, 2.77]	1.90 [1.40, 2.70]	2.00 [1.00, 3.00]	2.20 [1.55, 3.25]	< 0.001
Urea nitrogen, mg/dL	24.50 [15.00, 41.50]	19.50 [13.50, 32.50]	27.00 [16.00, 43.50]	18.32 [13.44, 26.97]	< 0.001
Anion gap, mEq/L	14.00 [12.00, 16.33]	14.00 [12.00, 16.50]	10.67 [8.00, 14.00]	12.40 [10.17, 15.30]	< 0.001
INR	1.33 [1.20, 1.68]	1.30 [1.20, 1.53]	NA [NA, NA]	1.21 [1.11, 1.33]	< 0.001
PTT, sec	33.00 [28.10, 42.63]	30.75 [27.50, 37.15]	35.00 [30.00, 44.00]	29.70 [26.20, 34.05]	< 0.001
Platelet count, K/μL	198.00 [135.58, 277.00]	160.33 [117.67, 222.67]	173.00 [120.50, 241.00]	194.75 [153.62, 240.92]	< 0.001
SOFA	6.00 [4.00, 9.00]	5.00 [4.00, 8.00]	4.00 [3.00, 6.00]	10.00 [8.00, 11.00]	< 0.001
Mortality, n	19.00 [17.00, 22.00]	19.00 [17.00, 22.00]	20.00 [17.00, 23.00]	17.77 [15.61, 19.65]	< 0.001

Data are median [IQR] or N (%). *P* value: continuous variables were assessed by the Kruskal Wallis rank sum test, and categorical variables were evaluated by the Chi-square test

*INR* international normalized ratio, *SysBP* systolic blood pressure, *PTT* activated partial thromboplastin time

**Table 2:** Characteristics of survivors versus non-survivors in the training set

Characteristic	Survival	Non survival	*P* value
Number of patients	14,001	2,740	
Age	67.00 [54.00, 79.00]	72.00 [59.00, 82.00]	< 0.001
Gender			
Male	7,819 (55.9)	1,524 (55.7)	0.854
Female	6,182 (44.1)	1,216 (44.3)	
Respiratory rate, breaths/min	19.00 [16.00, 22.00]	21.00 [18.00, 25.00]	< 0.001
Spo2, %	98.00 [96.00, 99.00]	97 [95.00, 99.00]	< 0.001
Temperature, °C	36.87 [36.45, 37.35]	36.72 [36.13, 37.31]	< 0.001
Heart rate, beats/min	87.00 [76.00, 98.00]	92.00 [79.00, 105.00]	< 0.001
DiasBP, mmHg	114 [105.00, 127.00]	108.00 [99.00, 120.00]	< 0.001
Creatinine, mg/dL	1.10 [0.75, 1.80]	1.47 [0.90, 2.57]	< 0.001
Urine output, ml	1560.00 [925.00, 2435.00]	905.00 [353.00, 1631.00]	< 0.001
Lactate, mmol/L	1.70 [1.20, 2.47]	2.50 [1.60, 4.58]	< 0.001
Urea nitrogen, mg/dL	23.00 [14.50, 38.50]	34.00 [20.00, 55.55]	< 0.001
Anion gap, mEq/L	13.67 [12.00, 16.00]	16.00 [13.00, 19.33]	< 0.001
INR	1.30 [1.20, 1.60]	1.50 [1.23, 2.10]	< 0.001
PTT, sec	32.50 [27.90, 40.64]	37.73 [29.53, 52.75]	< 0.001
Platelet count, K/μL	201.00 [140.67, 279.00]	180.50 [106.00, 270.00]	< 0.001
SOFA	6.00 [4.00, 9.00]	10.00[7.00, 14.00]	< 0.001

Data are median [IQR] or N (%). *P* value: continuous variables were assessed by the Kruskal Wallis rank sum test, and categorical variables were evaluated by the Chi-square test

*INR* international normalized ratio, *SysBP* systolic blood pressure, *PTT* activated partial thromboplastin time

### Risk factors analysis

**Fig. 2:**
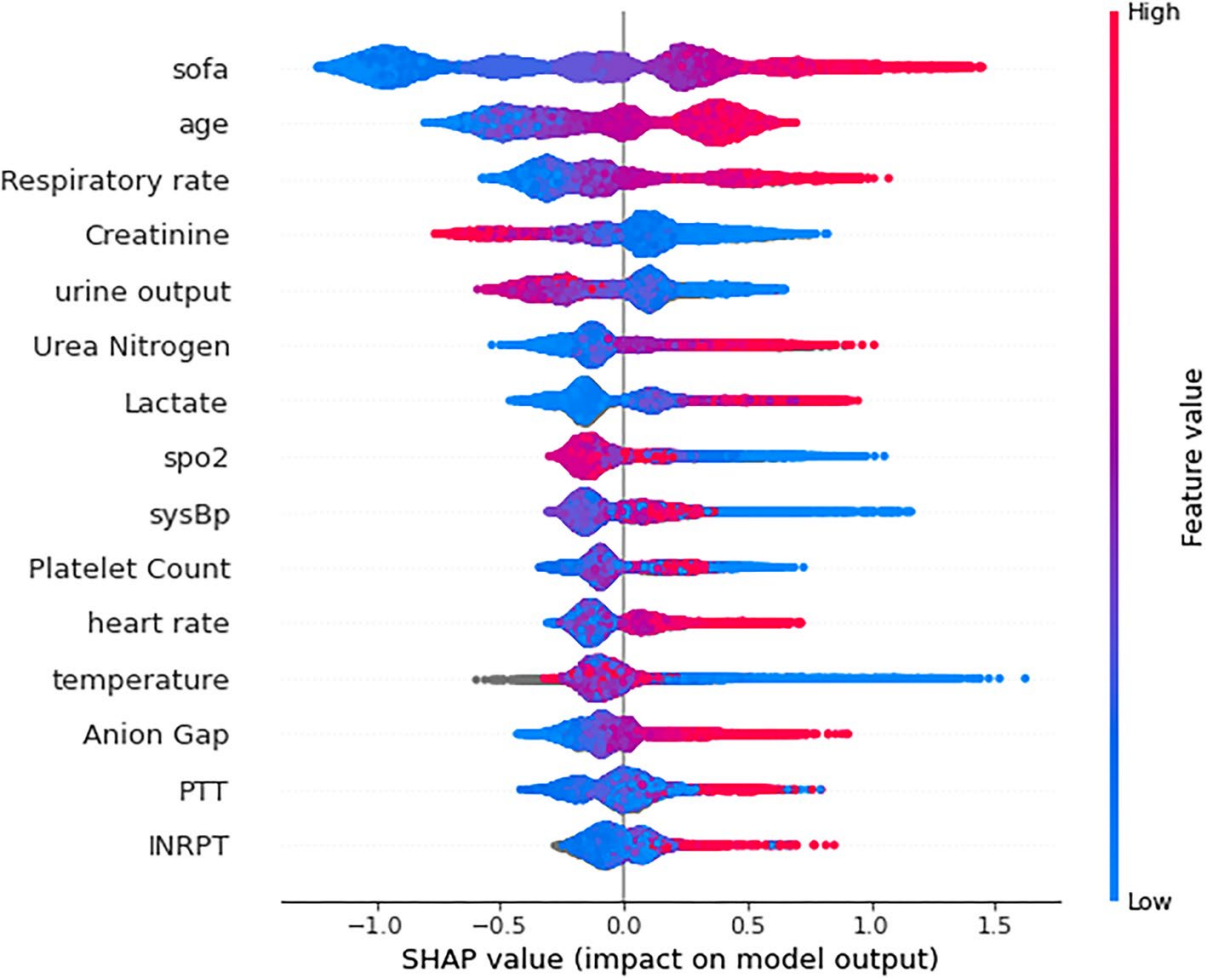
SHAP summary plot of the proposed model

Although the number of features is very important for the model training, increasing the number of features can also increase the difficulty and cost. Feature selection methods can be used to reduce the number of useless features, thus reducing the complexity of the final model [38]. Several common feature attribution methods for XGBoost model are inconsistent and may prevent the meaningful comparison of feature attribution values, whereas SHAP values can better explain the impact and importance of individual feature [25]. The significance of each feature was sorted according to the SHAP values. Following selection, the 15 features with the highest contribution were used to construct the final model, as shown in Fig. 2.

**Fig. 3:**
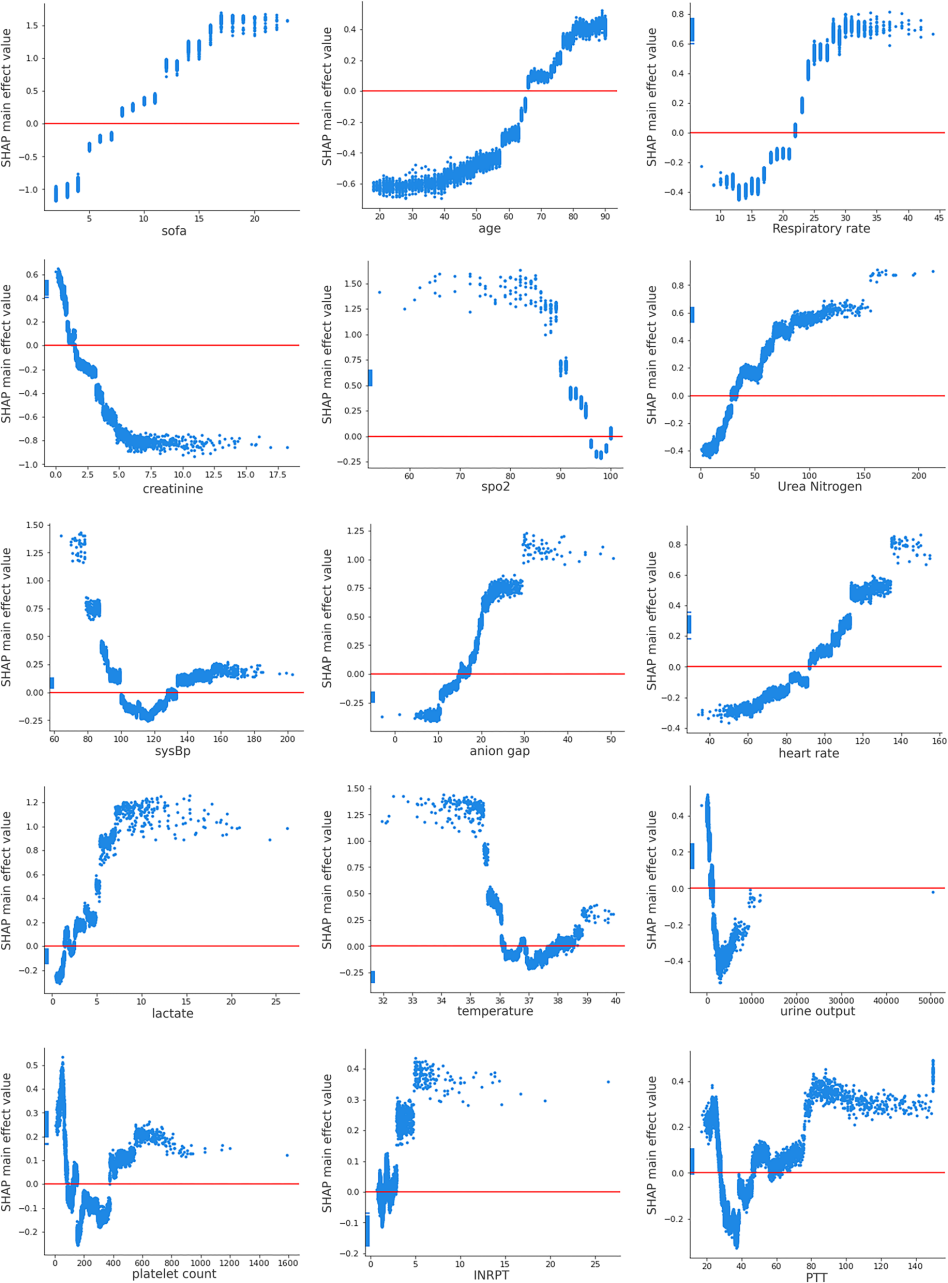
SHAP dependency plots of the top 15 features. The X-axis represents the actual value of the feature, the Y-axis represents the SHAP value of the feature, and the points correspond to the samples in the training set. A SHAP value above zero indicates an increased risk of in-ICU mortality

Furthermore, as shown in Fig. 3, the SHAP dependency plots for the contributing features quantified the clinical relationship between the in-ICU mortality and risk factors. The SHAP value is a measure of feature importance, with a higher value suggesting a greater influence on the increased risk of death. The approximate thresholds leading to the increased mortality were, SOFA score > 7 points, age > 65 years, respiratory rate > 22 breaths/min, serum creatinine < 2.0 mg/dL, urea nitrogen > 35 mg/dL, mean systolic blood pressure < 90 mmHg or > 130 mmHg, anion gap > 16 mEq/L, mean heart rate > 95 beats/min, serum lactate > 2.5 mmol/L, and platelet count < 150 K/μL or > 380 K/μL.

### Performance comparison among machine learning methods

**Fig. 4:**
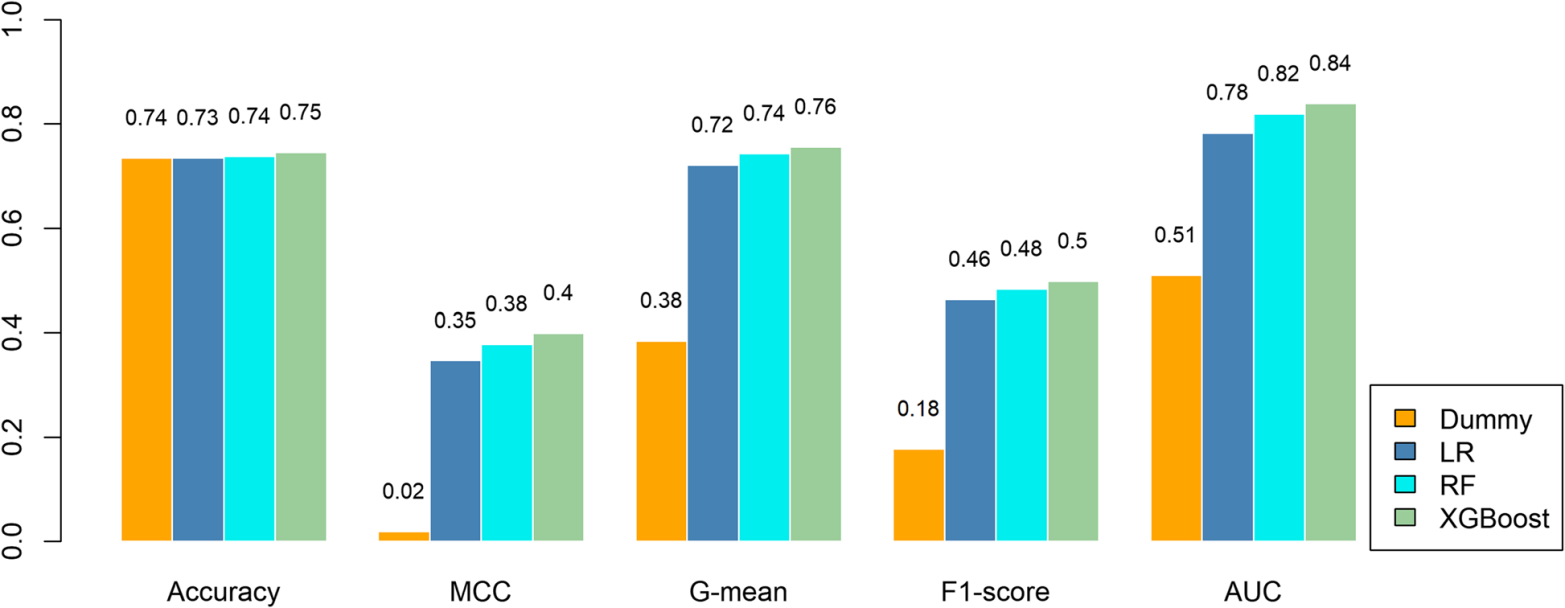
Performance evaluation of different ML methods

A comprehensive summary of the performance for each classifier during model development is shown in Fig. 4. All methods performed better than the dummy classifier, and XGBoost had relatively better performance compared with other ML methods.

### Cross-validation

**Fig. 5:**
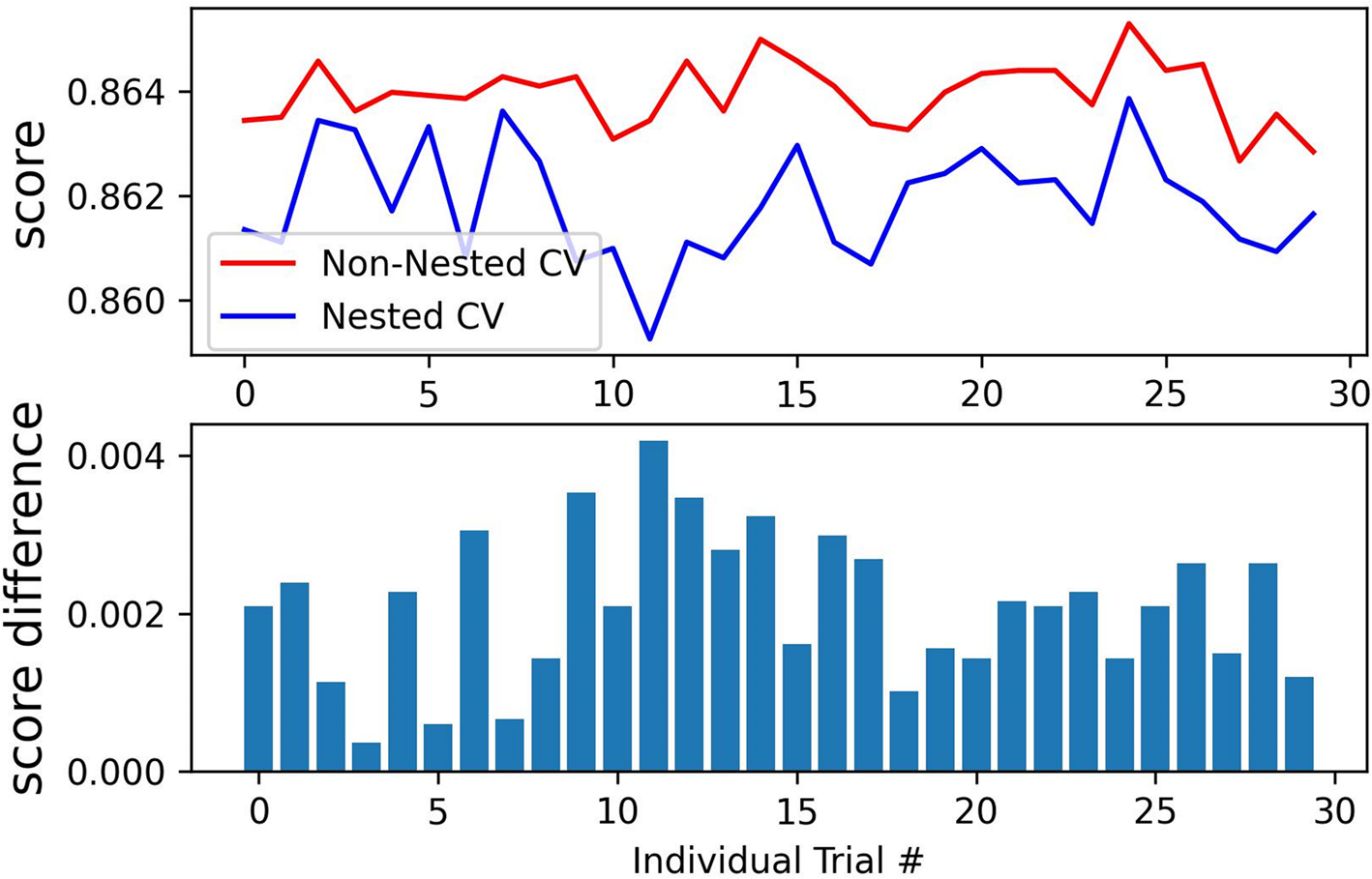
Non-nested and nested cross-validation on XGBoost in training dataset

The results from nested and non-nested CV on the training dataset are compared in Fig. 5. Thirty trials were conducted. Then, the CV scores and the average difference in scores from each trial were calculated. The average difference was 0.002086 with standard deviation of 0.000918. The non-nested CV scores were slightly higher than the nested CV scores.

### Performance comparison with clinical scores

**Fig. 6:**
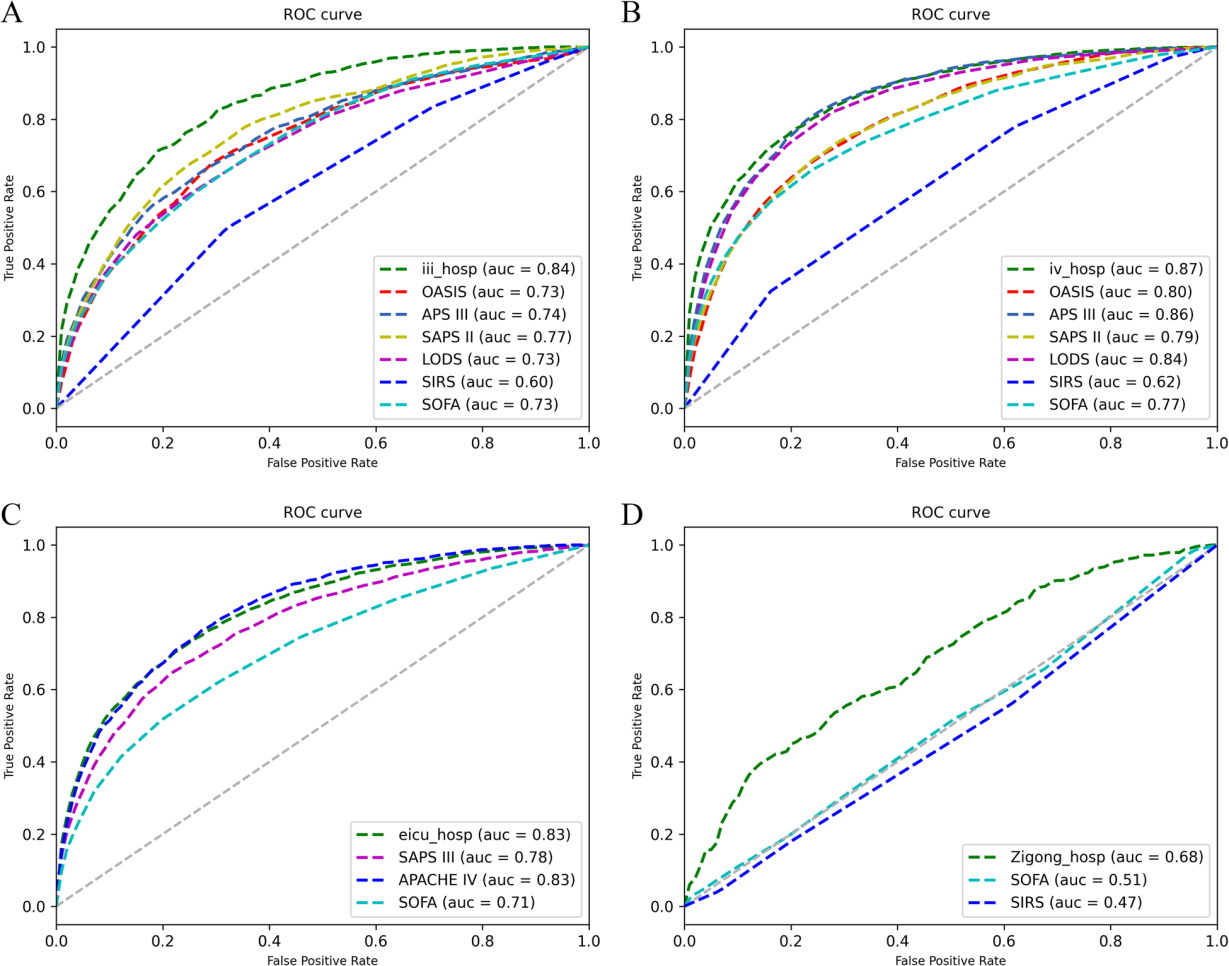
The area under the receiver operating characteristic (AUROC) curve. Internal validation performance: **A** The new prediction model in the MIMIC-III population during ICU hospitalization (iii_hosp) versus the OASIS, APACH III, SAPS II, LODS, SIRS, and SOFA models. External validation performance: **B** The new model used in the MIMIC-IV population (iv_hosp) versus the OASIS, APACH III, SAPS II, LODS, SIRS, and SOFA systems. **C** The new model used in the eICU population (eicu_hosp) versus the SAPS III, APACH IV, and SOFA scores. **D** The new model used in the Zigong population (Zigong_hosp) versus the SOFA and SIRS scores

The AUROCs of the proposed model and clinical scores in validation cohorts are provided in Fig. 6. The Delong test results were also assessed. Compared with the traditional scoring systems, the proposed model had better or equivalent performance. The Delong test also revealed that there were significant differences in AUCs between the present model and the traditional scoring systems (Delong test: *p* < 0.001), except for the APACH IV (Delong test: *p* = 0.4364) developed from the eICU database.

### Decision curve analysis

**Fig. 7:**
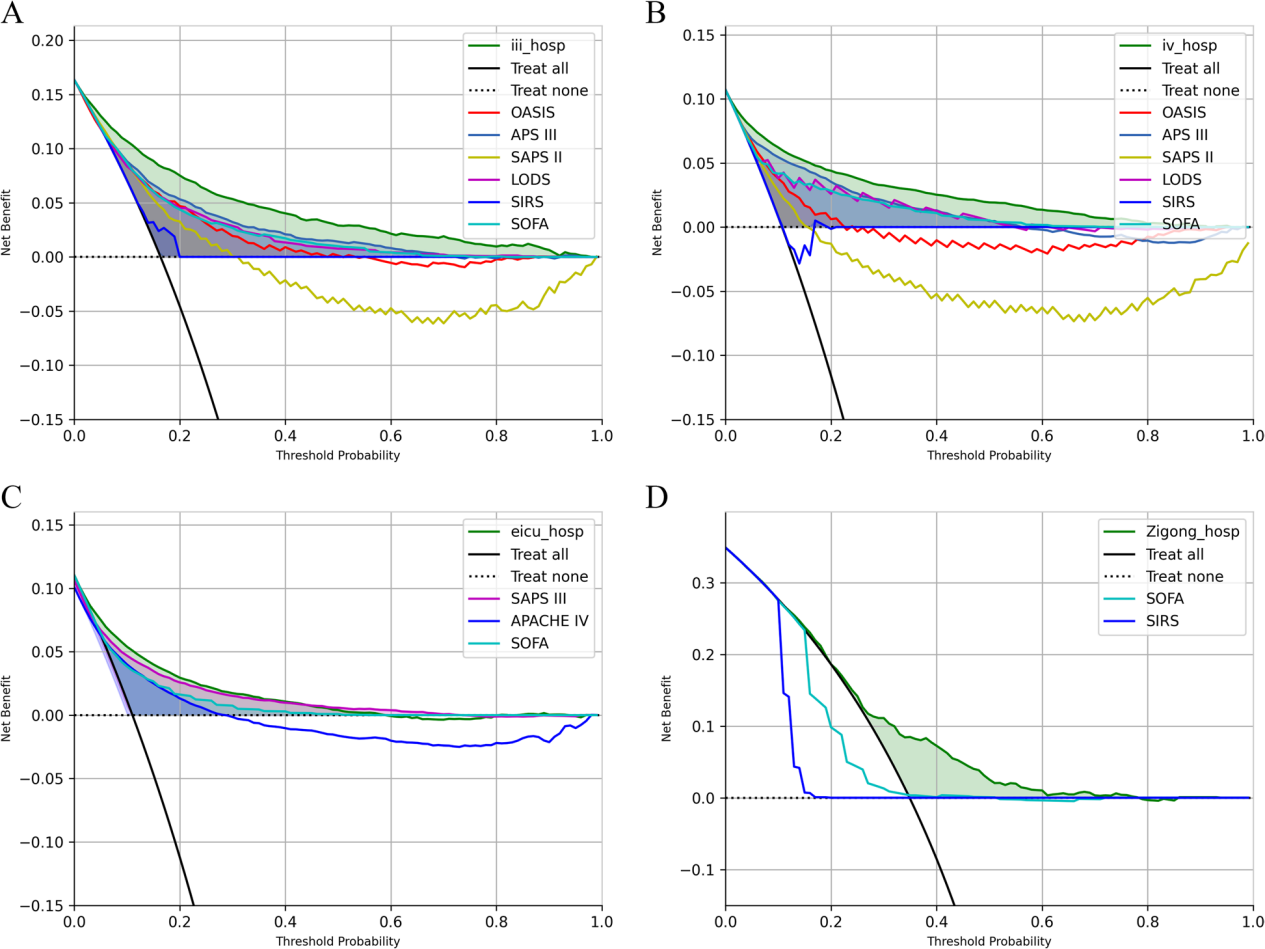
Decision curve analysis of proposed model and traditional scoring systems in four populations: **A** MIMIC-III population; **B** MIMIC-IV population; **C** eICU population; **D** Zigong population

The DCAs of the proposed model and traditional scoring systems in four populations are provided in Fig. 7. DCA graphically presents the “net benefit” obtained by applying each strategy [39]. In this analysis, for all databases, the net benefit of the proposed model was better than the net benefit of any of the traditional scoring systems.

### Discrimination and calibration

To better compare the discriminative abilities of the model, the whole population was grouped into five levels according to the predicted probability of in-ICU mortality of sepsis patients. The mortality of each level was calculated as the number of deaths in the level divided by the total number of patients in the level. As shown in Table 3, the relative rate of patient mortality was consistent with risk score, suggesting that the proposed model had a good risk stratification power and could successfully identify the mortality risk of patients.

**Table 3:** Relative risk ratios for various in-ICU mortality risk groups in different populations

**Risk**	**MIMIC-III**			**MIMIC-IV**		
	**Patients, N**	**Mortality, %**	**RR**	**Patients, N**	**Mortality, %**	**RR**
**> 80%**	149	83.89	5.13	334	82.63	7.71
**60–79%**	189	63.49	3.88	414	62.08	5.79
**40–59%**	321	46.73	2.86	602	40.20	3.75
**20–39%**	708	29.1	1.78	1404	22.93	3.75
**< 19%**	4158	7.29	0.45	12,778	4.45	0.41
**Total**	5525	16.36	-	15,532	10.72	-
**Risk**	**eICU**			**Zigong**		
	**Patients, N**	**Mortality, %**	**RR**	**Patients, N**	**Mortality, %**	**RR**
**> 80%**	203	82.76	7.49	30	76.67	2.17
**60–79%**	473	70.19	6.36	158	56.96	1.61
**40–59%**	820	44.02	3.99	333	43.84	1.24
**20–39%**	2329	23.66	2.14	567	27.41	0.78
**< 19%**	18,792	5.78	0.52	210	17.14	0.49
**Total**	22,617	11.04	-	1198	35.31	-

*RR* Relative risk ratios

**Fig. 8:**
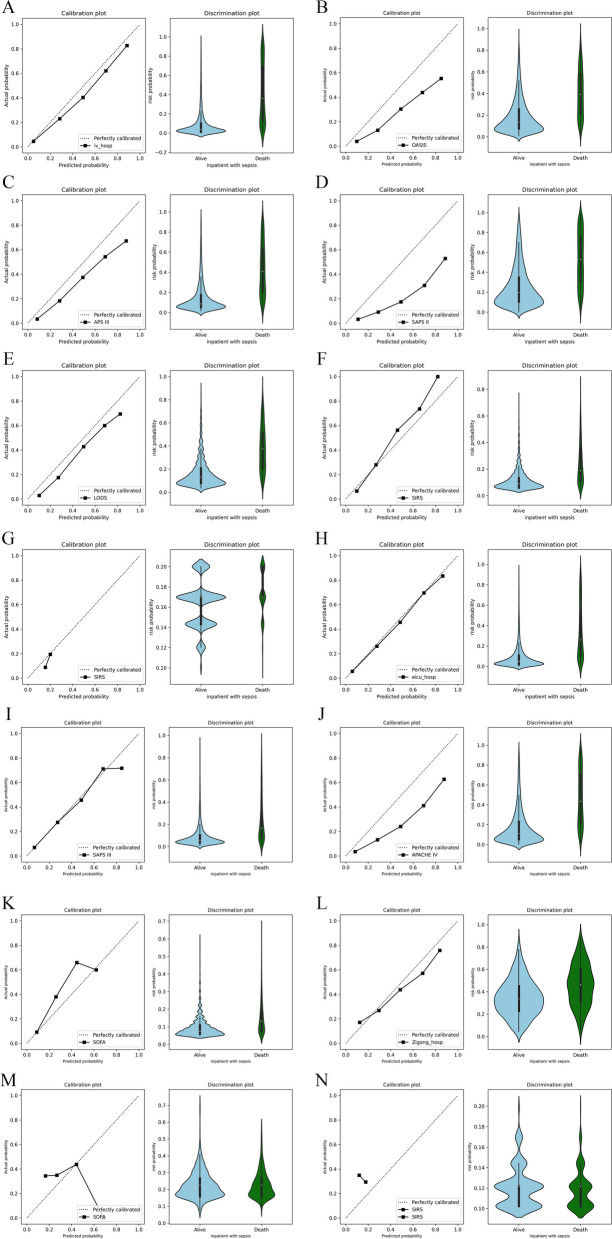
Calibration and discrimination potentials of the proposed model and traditional scoring systems in external validation. **A**–**G** The external validation results in the MIMIC-IV dataset: **A** The new model (Brier score = 0.068; C-index = 0.862; discrimination slope = 0.303); **B** OASIS (Brier score = 0.097; C-index = 0.795; discrimination slope = 0.215); **C** APS III (Brier score = 0.079; C-index = 0.857; discrimination slope = 0.287); **D** SAPS II score (Brier score = 0.126; C-index = 0.794; discrimination slope = 0.275); **E** LODS score (Brier score = 0.082; C-index = 0.844; discrimination slope = 0.222); **F** SOFA score (Brier score = 0.0821; C-index = 0.773; discrimination slope = 0.123); **G** SIRS score (Brier score = 0.097; C-index = 0.621; discrimination slope = 0.011). **H**–**K** The external validation results in the eICU dataset: **H** The new model (Brier score = 0.083; C-index = 0.820; discrimination slope = 0.270); **I** SAPS III (Brier score = 0.081; C-index = 0.782; discrimination slope = 0.154); **J** APACHE IV score (Brier score = 0.091; C-index = 0.826; discrimination slope = 0.290); **K** SOFA score (Brier score = 0.090; C-index = 0.714; discrimination slope = 0.060). **L**–**N** The external validation results in the Zigong dataset: **L** The new model (Brier score = 0.210; C-index = 0.679; discrimination slope = 0.123); **M** SOFA score (Brier score = 0.250; C-index = 0.505; discrimination slope = 0.003); **N** SIRS score (Brier score = 0.285; C-index = 0.469; discrimination slope = -0.002)

The differences between the predicted probabilities of in-ICU mortality among the survivors and non-survivors provided from each model were used to evaluate the discrimination (Fig. 8). Among the proposed model and traditional scoring systems, the proposed model had the best discrimination in MIMIC-IV and Zigong populations, with a discrimination slope of 0.303 and a c-index of 0.862 in MIMIC-IV, and a discrimination slope of 0.123 and a c-index of 0.679 in Zigong. The violin plots suggested that the new model can focus on the true negative while ensuring the stability of true positives in the risk distribution.

The proposed model exhibited excellent calibration properties (Fig. 8). The Brier scores of the proposed model were 0.068 (< 0.25) in the MIMIC-IV dataset, 0.083 (< 0.25) in the eICU dataset, and 0.210 (< 0.25) in the Zigong dataset. The Brier scores obtained by the new model were all less than 0.25, which meant that the model’s predictions were correct [40]. In comparison, traditional scoring systems were lacking in fitness and stability for the sepsis population. The calibration plots of the proposed model indicated good agreement between prediction and true outcome in the validation cohorts. The proposed model performed better across the entire range of mortality.

As shown in Table 4, in the MIMIC-IV population, among the proposed model and traditional scoring systems, the proposed model had the best discrimination, with a discrimination slope of 0.303 and a c-index of 0.862. The Brier score is a commonly used metric that measures the overall performance of the model [41]. Traditional scoring systems were lacking in fitness and stability for the sepsis population. The Brier scores of the proposed model were 0.0678 (< 0.25) in the MIMIC-IV and 0.0827 (< 0.25) in the eICU. However, APACHE IV had slightly better discrimination than the proposed model in the eICU population.

**Table 4:** Reclassification table

**External Validation**	**Initial Model**		**Predicted probability according to initial model**			**Reclassified (%)**	**Statistics**	
			< 5%	5–42%	> 42%		**NRI (95% CI)**	***p*-value**
**MIMIC-IV**	OASIS	< 5%	1026	807	166	49	0.789 (0.754–0.825)	< 0.0001
		5–42%	5592	4400	929	61		
		> 42%	1185	837	190	91		
	APS III	< 5%	1645	1193	253	47	0.773 (0.738–0.807)	< 0.0001
		5–42%	5682	4217	896	61		
		> 42%	876	634	136	92		
	SAPS II	< 5%	604	474	87	48	0.747 (0.710–0.784)	< 0.0001
		5–42%	5749	4194	897	61		
		> 42%	1850	1376	301	91		
	LODS	< 5%	463	340	60	46	0.830 (0.796–0.864)	< 0.0001
		5–42%	6959	5138	1089	61		
		> 42%	781	566	136	91		
	SIRS	< 5%	0	0	0	-	0.909 (0.876–0.941)	< 0.0001
		5–42%	8203	6044	1285	61		
		> 42%	0	0	0	-		
	SOFA	< 5%	893	663	151	48	0.848 (0.815–0.881)	< 0.0001
		5–42%	7125	5229	1101	61		
		> 42%	185	152	33	91		
**eICU**	SAPS III	< 5%	3649	3776	490	54	0.571 (0.542–0.600)	< 0.0001
		5–42%	5303	5412	690	53		
		> 42%	365	351	43	94		
	APACHE IV	< 5%	1824	1918	247	54	0.560 (0.526–0.594)	< 0.0001
		5–42%	5502	5664	733	52		
		> 42%	1213	1185	159	94		
	SOFA	< 5%	1822	1781	239	53	0.636 (0.609–0.663)	< 0.0001
		5–42%	8776	8776	1141	53		
		> 42%	30	46	6	93		
**Zigong**	SIRS	< 5%	0	0	0	-	0.318 (0.258–0.379)	< 0.001
		5–42%	29	690	479	42		
		> 42%	0	0	0	-		
	SOFA	< 5%	0	0	0	-	0.304 (0.244–0.365)	< 0.0001
		5–42%	29	687	459	42		
		> 42%	0	3	20	13		

*NRI* net reclassification improvement, *OASIS* Oxford acute severity of illness score, *APS III* Acute Physiology Score III, *APACHE IV* Acute Physiology, Age, Chronic Health Evaluation IV

### Reclassification

In the external validations, we calculated the risk of each individual and divided all patients into three groups based on the risk cut-off at 95% sensitivity and 95% specificity [35]. The proposed model was considered as the updated model, and the traditional scoring systems were considered as the initial models. The net reclassification improvement (NRI) was calculated in Table 4. The proposed model reclassified a large proportion of patients, especially patients with predicted probability less than 42% according to the initial model.

The cNRI and IDI were also calculated and shown in Table 5. All results of cNRI and IDI were positive values, indicating that the proposed model had better discriminative ability than the traditional scoring systems.

**Table 5:** Reclassification statistics

Initial Model		MIMIC-IV	eICU	Zigong
OASIS	cNRI	0.964 (0.918–1.011)	-	
	IDI	0.330 (0.313–0.348)	-	
APS III	cNRI	0.960 (0.916–1.005)	-	
	IDI	0.329 (0.312–0.347)	-	
SAPS II	cNRI	0.893 (0.844–0.941)	-	
	IDI	0.322 (0.303–0.341)	-	
LODS	cNRI	1.019 (0.973–1.065)	-	
	IDI	0.331 (0.314–0.347)	-	
SIRS	cNRI	1.101 (1.056–1.146)	-	0.137 (0.078–0.195)
	IDI	0.330 (0.315–0.345)	-	0.126 (0.102–0.149)
SOFA	cNRI	1.053 (1.010–1.095)	0.896 (0.860–0.931)	0.285 (0.198–0.371)
	IDI	0.329 (0.313–0.344)	0.237 (0.226–0.247)	0.120 (0.098–0.142)
SAPS III	cNRI		0.780 (0.744–0.817)	
	IDI		0.238 (0.226–0.249)	
APACHE IV	cNRI		0.717 (0.673–0.761)	
	IDI		0.233 (0.218–0.249)	

*cNRI* continuous net reclassification index, *IDI* integrated discrimination improvement, *OASIS* Oxford acute severity of illness score, *APS III* Acute Physiology Score III, *APACHE IV* Acute Physiology, Age, Chronic Health Evaluation IV

## Discussion

The global burden and mortality caused by sepsis are greater than previously estimated, requiring urgent attention [1]. Sepsis associated deaths are potentially preventable with better hospital-based care [42]. ML techniques could help to develop risk stratification models to better manage the sepsis patients, including sepsis prediction [32, 43] and severity assessment [22, 23]. Therefore, the present study established and validated a predictive model based on the XGBoost algorithm for the risk stratification of sepsis patients in the ICU. The key findings and limitations of this study are discussed below.

There were many previous ML models to predict the mortality in sepsis patients, such as RF, gradient boosting decision tree (GBDT), and LR [22, 44], but few of them were widely used in the clinic. One major issue with these ML models was the black box effect, which made it hard to understand and interpret the selected features in the models. SHAP methodology that uses a game theoretic approach, was used for feature identification and prioritization, and it was also successfully used during the ML model construction in the clinic [23]. In addition to using SHAP to quantify the magnitude of contribution from each feature, we applied the XGBoost algorithm to create the ML model. The XGBoost algorithm uses a supervised gradient boosting approach for sequential model testing and selection. It has the advantages of excellent performance, automatic modeling of non-linearities and high-order interaction, and robustness to multicollinearity [45]. There were few recent studies that used SHAP and XGBoost to create the sepsis mortality prediction models [21, 23]. However, these studies either had no validation or validated the model in the same dataset to create the model. In the present study, we externally validated our model not only in a database in US but also in another database in China that was completely independent from the database used to develop the model. These external validations showed satisfactory generalizability and robustness of our proposed model.

Regarding feature selection, four large ICU databases were used in this retrospective cohort study. SHAP values were calculated to illustrate the contribution of each feature to the prediction task of the proposed model. The selected clinical features were highly consistent with clinical practice, especially with the SOFA, SIRS, and qSOFA scores. Clinical indicators such as serum lactate, heart rate, respiratory rate, temperature, white blood cell count, and platelet count are mentioned in sepsis-related guidelines, such as sepsis 3.0 [4], sepsis 1.0 [9], and sepsis-induced coagulopathy [46]. Correlations and changes among features were ignored due to the fractionalization process based on the features. New features were required to link to the in-ICU mortality in sepsis patients. An unexpected discovery of this study was that the relationship between serum creatinine (SCr) level and the mortality of patients with sepsis was contrary to the guideline from the Kidney Disease: Improving Global Outcomes [47]. However, some studies have reached conclusions similar to ours, namely, low admission SCr level and decrease in SCr after admission were associated with increased mortality [48, 49]. Sepsis and other complex problems may affect SCr generation [50]. In our study, the proposed model could explore the interaction among features and higher-order information that could potentially be helpful for risk assessment.

In the model development, XGBoost showed the highest quality of binary classification in imbalanced data compared to other algorithms.Additionally, there was not much difference between nested and non-nested CV, indicating that the non-nested approach could get good generalization and less intensive computational burden [51]. Regarding the performance of the proposed model with clinical scores, the results of this study demonstrated that the XGBoost algorithm could outperform the traditional scoring systems. DCA indicated that the proposed model obtained the best net benefit. Furthermore, the proposed model showed satisfactory discriminatory power and calibrated capacity. Both internal and external validations were done in both single- and multi-center databases. The proposed model yielded good performance in not only the internal validation but also external validations on three other databases. Thus, the proposed model has good generalizability and efficiency. Furthermore, among the traditional scoring systems, SIRS was not applicable to assess the risk of death of sepsis patients in these databases, and SOFA was greatly affected by the heterogeneity among the databases.

There were some limitations in this study. Firstly, the specific mechanism of clinical features in model construction was not clear. The SHAP method interpretsthe model well, and the background dataset’s choice can significantly impact the SHAP values, which may potentially lead to biased or incorrect interpretations [52], so further clinical verification is still needed. Additionally, due to the availability of databases, there was no way to compare the proposed model with every traditional scoring system in every database. Furthermore, assuming the baseline SOFA score of zero may result in the inclusion of patients with no change in SOFA score. Some clinical indicators that may be important for sepsis mortality prediction were missing. The validation of the model on the Zigong database was not satisfactory, probably due to the quality and heterogeneity of the data itself. False survivors, who chose to withdraw from treatment and died shortly after discharge [31], influenced the mortality calculation. In addition, potential selection bias may exist. Finally, the proposed model only used the data during the first 24 h after the ICU admission. Dynamic monitoring and prediction remain to be explored.

## Conclusions

In this study, a multi-source data-driven predictive model using the XGBoost algorithm was developed to predict the in-ICU mortality of patients with sepsis. The model not only showed significant improvement over current scoring systems (including SOFA, OASIS, APS III, SIRS, SAPS II, and SAPS III) but also revealed the associations between several clinical indicators and in-ICU mortality in sepsis patients. The results demonstrated the generalizability and robustness of the proposed model. Although further clinical validation is needed, the proposed model offers an example of how the application of ML based on multi-source data can be helpful for understanding disease mechanisms and optimizing clinical managements.

## Data Availability

The original databases of this article are available at https://physionet.org/content/mimiciii/1.4/, https://physionet.org/content/mimiciv/2.2/, https://physionet.org/content/eicu-crd/2.0/, and https://physionet.org/content/icu-infection-zigong-fourth/1.1/. The data processed and/or analyzed in this study are available from the corresponding author on reasonable request.

## Abbreviations

ICU: Intensive care unit
MIMIC: Medical Information Mart for Intensive Care
XGBoost: Extreme Gradient Boosting
SHAP: Shapley Additive Explanation
AUROC: Area under the receiver operating characteristic curve
SAPS: Simplified Acute Physiology Score
SOFA: Sequential Organ Failure Assessment
APACHE IV: Acute Physiology and Chronic Health Evaluation IV
SIRS: Systemic inflammatory response syndrome
ML: Machine learning
SQL: Structured query language
MCC: Matthews correlation coefficient
G-mean: Geometric mean
RF: Random forest
LR: Logistic regression
LODS: Logistic Organ Dysfunction System score
qSOFA: Quick SOFA
ROC curve: Receiver operating characteristic curve
cNRI: Continuous net reclassification index
CV: Cross-validation
IDI: Integrated discrimination improvement
CI: Confidence interval
IQR: Interquartile range
INR: International normalized ratio
SysBP: Systolic blood pressure
PTT: Activated partial thromboplastin time
AUC: Area under the curve
DCA: Decision curve analysis
RR: Relative risk ratios
OASIS: Oxford acute severity of illness score
APS III: Acute Physiology Score III
NRI: Net reclassification improvement
SCr: Serum creatinine
